# Association of the Rheumatoid Arthritis Severity Variant rs26232 with the Invasive Activity of Synovial Fibroblasts

**DOI:** 10.3390/cells8101300

**Published:** 2019-10-22

**Authors:** Emma R Dorris, Eimear Linehan, Michelle Trenkmann, Douglas J Veale, Ursula Fearon, Anthony G. Wilson

**Affiliations:** 1 University College Dublin Centre for Arthritis Research, Conway Institute, University College Dublin, Dublin D04 W6F6, Ireland; linehae@tcd.ie (E.L.); michelle.trenkmann@nature.com (M.T.); douglas.veale@ucd.ie (D.J.V.); gerry.wilson@ucd.ie (A.G.W.); 2 Molecular Rheumatology, School of Medicine, Trinity Biomedical Sciences Institute, Trinity College Dublin, Dublin D06 R590, Ireland; fearonu@tcd.ie

**Keywords:** rheumatoid arthritis, synovium, fibroblasts, genetics, C5orf30, genotype‒phenotype

## Abstract

rs26232, located in intron one of *C5orf30*, is associated with the susceptibility to and severity of rheumatoid arthritis (RA). Here, we investigate the relationship between this variant and the biological activities of rheumatoid arthritis synovial fibroblasts (RASFs). RASFs were isolated from the knee joints of 33 RA patients. The rs26232 genotype was determined and cellular migration, invasion, and apoptosis were compared using in vitro techniques. The production of adhesion molecules, chemokines, and proteases was measured by ELISA or flow cytometry. Cohort genotypes were CC *n* = 16; CT *n* = 14; TT *n* = 3. In comparison with the RASFs of the CT genotype, the CC genotype showed a 1.48-fold greater invasiveness in vitro (*p* = 0.02), 1.6-fold higher expression intracellular adhesion molecule (ICAM)-1 (*p* = 0.001), and 5-fold IFN-γ inducible protein-10 (IP-10) (*p* = 0.01). There was no association of the rs26232 genotype with the expression levels of either total C5orf30 mRNA or any of the three transcript variants. The rs26232 C allele, which has previously been associated with both the risk and severity of RA, is associated with greater invasive activity of RASFs in vitro, and with higher expression of ICAM-1 and IP-10. In resting RASFs, rs26232 is not a quantitative trait locus for C5orf30 mRNA, indicating a more complex mechanism underlying the genotype‒phenotype relationship.

## 1. Introduction

Genetic variants in *C5orf30* have been associated with the risk of several autoimmune diseases, including rheumatoid arthritis (RA) [1,2]. The rs26232 variant, located in the first intron, is also associated with the severity of radiological damage in RA [3]. The gene encodes a 206 amino acid protein that is only present in the vertebrae and is highly conserved across species. The expression of C5orf30 is highest in RA synovial fibroblasts (RASFs) and macrophages, key mediators of inflammation and tissue damage in RA [4]. Inhibition of C5orf30 in RASFs results in increased invasive and migratory activities in vitro, and in the collagen-induced arthritis model results in more severe inflammation and joint damage [5]. In macrophages, proinflammatory signals such as tumour necrosis factor (TNF) and lipopolysaccharide (LPS) reduce C5orf30 expression via a c-Jun N-terminal kinase mediated mechanism; furthermore, C5orf30 enhances the resolution of inflammation and wound repair functions mediated by M2 macrophages [6]. 

Most genetic variants associated with RA risk have relatively modest effects on gene expression in a restricted number of cell types: an RA-associated variant in *TRAF1* is associated with approximately 2-fold lower expression of TRAF1 in CD8 T cells and macrophages, with consequent higher production of pro-inflammatory cytokines including TNF and interleukin (IL)-6 [7]; a polymorphism in the IL-6 promoter, that has been associated with juvenile idiopathic arthritis, is an expression quantitative trait locus (eQTL) for IL-6 in synovial fibroblasts but not CD14+ monocytes [8]. These data highlight that genotype‒phenotype associations may be cell-type-specific.

The biological mechanisms underlying the genetic associations of rs26232 with RA risk and severity are unknown. We hypothesised that, given the effects of C5orf30 on RASF biology, the association of rs26232 arises from allelic-associated differences in the tissue-damaging activities of RASFs. We tested this hypothesis using RASFs from knee joints, using in vitro assays to compare these activities in cells of differing rs26232 genotypes.

## 2. Materials and Methods

### 2.1. Isolation and Culture of RASF

Synovial biopsies were obtained from the knee joints of 44 RA donors. Two donors (both TT genotype) could not be used in functional assays due to low density and slow doubling time. Fresh synovial biopsies were digested using 1 mg/mL collagenase type 1, as previously described [5]. Dissociated cells were grown to confluence in RPMI1640 medium (Lonza, Basel, Switzerland supplemented with 10% (*v*/*v*) heat-inactivated FBS, penicillin (100 units/mL), and streptomycin (100 units/mL) before trypsinization and passage. Cells were used at passage 3 to ensure a homogeneous population of fibroblasts. 

### 2.2. rs26232 Genotyping

A TaqMan^®^ SNP Genotyping Assay was used to determine rs26232 genotype, as previously described [9]. Genotyping reactions were run on a QuantStudio7 Flex Real-Time PCR System (Applied Biosystems, Waltham, MA, USA) using standard cycling conditions and a post-read allelic discrimination assay. All samples were run in technical duplicate with positive and no-template controls.

### 2.3. RASF Invasion 

In vitro invasion of RASF was assayed in a transwell system using the BD BioCoat Matrigel Invasion Chamber, as previously described [10]. Briefly, RASF were harvested by trypsin‒EDTA digestion; 2 × 10^4^ cells were resuspended in 500 μL of serum-free RPMI and plated in the upper compartment of the Matrigel-coated inserts. The lower compartment was filled with complete, supplemented media, and incubated at 37 °C for 24 h. The upper surface of the insert was cleaned with a cotton swab to remove noninvading cells and the Matrigel layer, as previously described. Insert were stained with 0.1% crystal violet. Five fields of invading cells were counted from a 10× objective magnification (100× total magnification): one random field from each quarter of the membrane and a central field. Cells from each field were added together to calculate the total number of invading cells. 

### 2.4. RASF Migration

In vitro invasion of RASF was assayed in a wound repair assay [10]. In 48-well plates, 2 × 10^4^ RASF were seeded per well in technical duplicate. Cells were grown for 48 h and a single scratch wound was induced through the middle of each well and imaged at 10× objective magnification (100× total magnification). After 18 h, wells were washed with PBS and fixed with 4% paraformaldehyde (PFA) prior to staining with crystal violet. Semi-quantitative analysis of cellular repopulation of the wound margins was performed by counting the number of cells migrating into the wound space. This process was repeated for all technical replicates, and these were averaged to determine the number of migrating cells per RASF donor.

### 2.5. RASF Proliferation 

RASF were seeded into 96-well plates at 5 × 10^3^ cells per well in technical triplicate. Cells were incubated in complete media at 37 °C for 4 h and 96 h. RASF were washed with PBS and fixed with 4% PFA prior to staining with crystal violet. Crystal violet was solubilized with 1% triton X and absorbance was measured at 570nm. Empty (background) well controls were subtracted from the optical density readings. Technical replicates were averaged to determine the number of migrating cells per RASF donor. Proliferation is reported as fold-change optical density at 96 h compared to the baseline (4 h).

### 2.6. Flow Cytometry

RASF were plated into six-well plates at 1 × 10^5^ cells per well in complete media. Cells were incubated at 37 °C for 48 h. RASF were incubated with 0.3 μg/mL of phycoerythrin-conjugated mouse monoclonal anti-intracellular adhesion molecule (ICAM)-1 or isotype-matched IgG control (all Becton Dickinson, Franklin Lakes, NJ, USA) for 30 min at 4 °C. Cells were then washed twice and fixed in 1% paraformaldehyde, and analysed by flow cytometry (FACS) using a FACScan flow cytometer and Lysis II software (both from Becton Dickinson). Adhesion marker expression was calculated based on the median fluorescence intensity (MFI), with background MFI (unstained samples) subtracted. Technical replicates were averaged to determine the MFI per RASF donor.

### 2.7. Cytokine and Protease Production

Cells (2 × 10^4^ per well) were cultured for 48 h and the supernatants harvested. Matrix Metalloproteinase 3 (MMP3) and Tissue Inhibitor of Metalloproteinase 3 (TIMP3) were measured by single-plex ELISA (R&D Systems, Minneapolis, MN, USA), IP10, monocyte chemoattractant protein 1 (MCP1) and macrophage inflammatory protein 1 alpha (MIP1α) were measured via multiplex ELISA (Meso-scale discovery, Rockville, MD, USA).

### 2.8. Real-Time PCR

Total mRNA was extracted from cultured RASF and reverse-transcribed. Reverse transcription of 100 ng of total mRNA was carried out using a high-capacity cDNA reverse transcription kit (Applied Biosystems, Waltham, MA, USA), as previously described [6]. Real-time PCR was carried out on a Roche Lightcycler (Basel, Switzerland) under standard cycling conditions. Levels of total C5orf30 mRNA and of each of the three 5′ untranslated transcript variants, variant-1, NM_001316968.1; variant-2, NM_033211.3; and variant-3, NM_001316969.1, were measured (Supplementary Table S1). The housekeeping gene *HPRT1* was used as an endogenous control. Relative quantitation (ΔΔCt method) of ICAM1, MMP14, CDH11, VCAM, and CTSK was carried out via TaqMan real-time PCR on an Applied Biosystems QuantStudio 7 Flex Real-Time PCR System using standard cycling conditions and 18s rRNA as an endogenous control. Assay IDs can be found in Table S2.

### 2.9. Data Analysis

All data were tested for parametric assumptions. Parametric data were analysed using a one-way ANOVA with Tukey’s post hoc test for honest significant difference. Non-parametric data were analysed using a Kruskal–Wallis test. An alpha level of 0.05 was used for all analyses. 

## 3. Results

### 3.1. Association of the rs26232 CC Genotype with Greater Invasiveness of RASFs In Vitro

**Figure 1:**
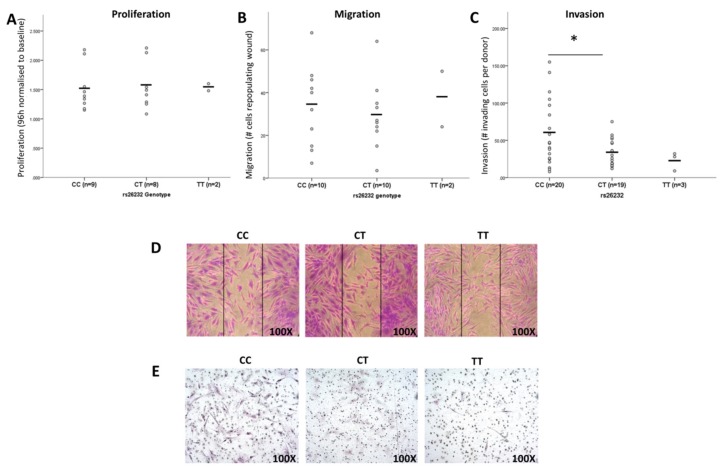
The rs26232 genotype is associated with RASF invasiveness. (**A**) Proliferation rates of RASFs are not associated with rs26232 genotype (*p* = 0.980). (**B**) The capacity for cells to migrate was not associated with genotype (*p* = 0.783). (**C**) RASF of the CC genotype are more invasive than CT (*p* = 0.020). Representative images of RASF in vitro (**D**) migration and (**E**) invasive assays of rs26232 genotypes. Each circle represents the average of an individual donor. Horizontal black bars represent the cohort mean. Statistical significance: * *p* < 0.05.

Synovial fibroblasts were isolated from 42 RA knee joints; the distribution of genotypes was CC, 20; CT, 19; TT, 3. In view of the low TT genotype frequency, comparisons were restricted to CC and CT genotypes. There was no difference in proliferation rate (*p* = 0.980) (Figure 1A), nor in the rate of cellular migration (*p* = 0.783) (Figure 1B). Invasive activity, as assessed using the Matrigel assay, was 1.9-fold higher in CC-genotype RASFs (*p* = 0.02) (Figure 1C). Representative images from each genotype are shown in Figure 1D,E.

### 3.2. Higher ICAM-1, MMP14, and IP-10 Production by RASFs of CC Compared to CT Genotype

**Figure 2:**
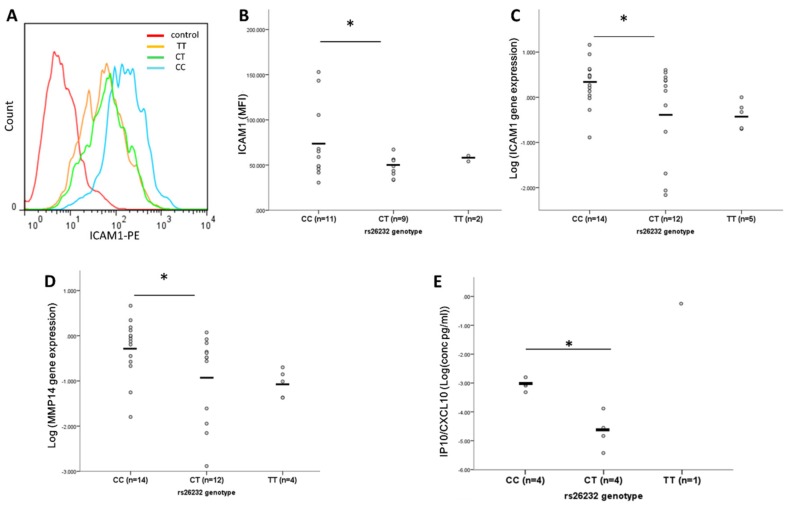
The rs26232 genotype is associated with RASF expression of ICAM1, *MMP14* and IP-10. (**A**) Representative histogram showing ICAM-1 expression by RASFs of different rs26232 genotypes. (**B**) Expression of ICAM-1 protein is greater in RASFs of the CC compared to CT genotype (1.5-fold, *p* = 0.039). (**C**) ICAM1 relative gene expression is higher in CC compared to CT RASFs (2-fold, *p* = 0.044). (**D**) MMP14 relative gene expression is higher in CC compared to CT RASFs (1.6-fold, *p* = 0.021) (**E**) RASFs of CC genotype produce greater IP10 (CXCL10) compared to CT genotype (5-fold, *p* = 0.011). Each circle represents an individual donor. The black bar represents the mean. Statistical significance: * *p* < 0.05.

The expression of ICAM-1 was compared between genotype groups using flow cytometry. The CC genotype was associated with 1.5-fold higher ICAM-1 expression (*p* = 0.039) compared with CT RASFs (*n* = 11 (CC), *n* = 9 (CT) for both groups, Figure 2A,B). Relative ICAM-1 gene expression was also higher in CC (*n* = 14) compared to CT (*n* = 12) RASFs (*p* = 0.44, Figure 2C), as was the relative gene expression of the matrix metalloprotease MMP14 (*p* = 0.021, Figure 2D). Basal production of the chemokine IP-10 was 5-fold greater in RASFs of the CC genotype compared with CT cells (*n* = 4 each, *p* = 0.011, Figure 2C). There was, however, no difference in the basal secretion of MMP3 (*p* = 0.457), TIMP3 (*p* = 0.374), MIP1 (*p* = 0.713) or MCP1 (*p* = 0.982) (Supplementary Figure S1A–D, respectively) between CC and CT RASFs (*n* = 4 for both groups). There was no observed difference in relative gene expression of the adhesion markers cadherin 11 (CDH11, *p* = 0.934) and vascular cell adhesion protein 1(VCAM, *p* = 0.509) nor the protease cathepsin K (CTSK, *p* = 0.349) (Figure S2A–C).

### 3.3. rs26232 Genotype Is Not Associated with C5orf30 mRNA Expression in RASFs

**Figure 3:**
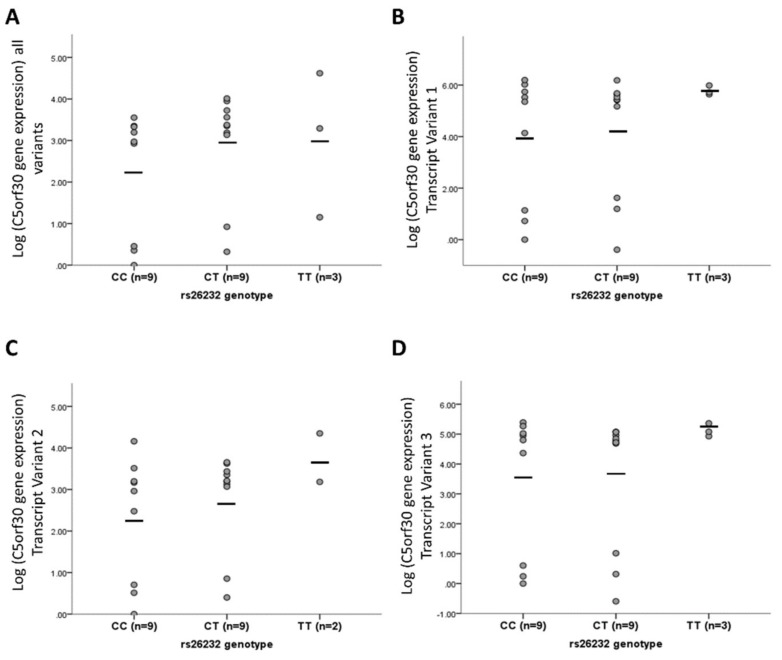
rs26232 is not an eQTL for C5orf30. rs26232 genotype was not associated with (**A**) total C5orf30 mRNA (all variants) (*p* = 0.506), (**B**) variant 1 (*p* = 0.469), (**C**) variant 2 (*p* = 0.352), or (**D**) variant 3 (*p* = 0.482). Gene expression is analysed using relative quantitation (RQ) to the endogenous control HPRT1. *Y*-axis is mean Log_2_ transformed RQ values per patient RASF line. Each circle represents the average of an individual donor. Horizontal black bars represent the cohort mean.

The expression of total mRNA and of each of the three 5′ variants was compared between the two rs26232 genotypes (*n* = 9, both groups). There was no difference in either total mRNA levels (*p* = 0.506), or for individual transcripts: variant 1 RefSeq NM 001316968.1 (*p* = 0.469), variant 2 NM 033211.3 (*p* = 0.352), or variant 3 NM 001316969.1 (*p* = 0.482) (Figure 3A–D, respectively).

## 4. Discussion

The C allele of rs26232 is both a risk and severity variant for RA; it has been linked to radiological damage in the joints of the hands and feet, with a per T allele effect on radiological damage score of 0.9 [3]. In the present study, we examined, using in vitro assays of biological activities, if genotype-phenotype correlations are present in RASFs. Our data reveal significant differences in the invasive activities of RASFs of different rs26232; CC RASFs being more invasive than CT. There were no genotype associations with other pathobiological characteristics of RASFs such as migration, apoptosis, or proliferation. The CC genotype was also associated with higher expression of the adhesion molecule ICAM-1, the membrane-type 1 metalloproteinase MMP14, and the T cell chemokine, IP-10. Notably, however, there was no significant quantitative or qualitative difference in the total C5orf30 mRNA with rs26232 genotype.

The RASF is a central mediator of cartilage damage, producing many disease-relevant effectors including chemokines, adhesion molecules, and proteases [11]; furthermore, it has a semi-transformed phenotype in vitro with loss of contact inhibition, high proliferative activity, and resistance to apoptosis [12,13]. Inhibition of C5orf30 upregulates the invasive activities of RASFs in vitro [5]. The invasive activities of RASFs in vitro have been shown to positively correlate with the rate of radiological damage in the joints of the hands and feet [14]. We have recently reported that RA synovial C5orf30 mRNA levels are inversely correlated with both systemic (DAS28-ESR) and intra-articular (TNF mRNA) measures of disease activity [6]. Our data suggest that the association of the rs26232 C variant with radiological damage is likely to be related to the higher invasive potential of RASFs. This association may be mechanistically related to the differential expression of several important disease mediators: increased ICAM-1 expression can potentiate interactions with T cells and, in turn, is important for T cell and fibroblast activation and associated with sustaining both the proinflammatory and invasive processes in the synovium [15,16,17]. IP-10 is produced by RASFs and synovial macrophages and is a chemoattractant for a range of innate and adaptive immune cells [18]. MMP14 has abundant expression in the RA synovium and can be produced by synovial fibroblasts and CD68-positive osteoclasts and macrophages [19,20]. MMP14 has also been found to be increased in pathogenic RA fibroblasts, associated with increased invasiveness [21]. Recent studies have increased our understanding of the heterogeneity of fibroblasts in joint tissue and the role of fibroblast subtypes involved in RA pathology [21,22]. We observed no association between *CD11* expression and the rs26232 genotype. However, the association between the rs26232 genotype and relative levels of fibroblast subtypes or fibroblast markers such as fibroblast activation protein-α (FAP), Thy-1 cell surface antigen (THY1), or podoplanin (PDPN) remains unknown. 

Most genetic variants linked with common illnesses, such as RA, are in regulatory regions and are eQTLs for one or more nearby genes. There was, however, no evidence that rs26232 is an eQTL for C5orf30 mRNA in RASF. There had been a report that C5orf30 3′ untranslated transcript variants’ levels are associated with the rs26232 genotype in peripheral blood leucocytes [23]; however, subsequent data indicated that only 5′ untranslated variants are present in humans. Our data reveal that rs26232 is not an eQTL for C5orf30 mRNA, or for individual transcript variants, in RASFs. In agreement with these data in RASFs, the Genotype-Tissue Expression Project (GTEx) database does not report rs26232 to be eQTL for C5orf30 in any tissue; however, it is for the nearby transcripts of *EIF3KP1*, *PAM*, and *PPIP5K2* in a limited number of tissues including the peripheral blood, skeletal muscle, and subcutaneous adipose tissue [24]. The basis of the rs26232 association with both RA radiological severity and RASF behaviour in vitro could arise from several other mechanisms; the eQTL for C5orf30 may only be manifest in the context of the complex inflammatory and hypoxic environment of the RA joint, or be restricted to a different cell type, or could be the result of altered expression of one of the nearby genes. Currently, there is no evidence implicating these loci in RA.

The functional consequences of variants occurring in untranslated regions of genes, such as rs26232, are frequently not immediately obvious. Data from ENCODE (the Encyclopaedia of DNA Elements) have increased our understanding of the impact that sequence variations have on gene expression, but the functional consequences of variants in the 5′ UTR still remain largely uncharacterized [25,26]. Annotating non-coding SNPs is more challenging as the consequences of variation are not as well understood [27]. However, a number of studies have shown that a single variant in the noncoding region can have dramatic effects on gene regulation via, for example, disruption of transcription factor binding and modification of chromatin accessibility [28,29,30,31]. Recently, it is being realized that regulatory variants can account for the major part of the genetic predisposition to complex traits [32]. In an extensive study of complex diseases including RA, regulatory variants, particularly those in DNase hypersensitivity regions, accounted for much more heritability than coding variants [33]. Rs262332 is predicted to be in a DNase hypersensitivity cluster (UCSC genome browser GRCh38/hg38: http://genome.ucsc.edu/) [34]. Although it is currently difficult to predict the consequences of 5′ UTR variants due to a lack of knowledge of RNA structural complexity and the RNA binding protein (RBP) landscape, data from large-scale cancer genome sequencing projects will likely lead to the uncovering of new biological mechanisms [26,35,36,37].

## 5. Conclusions

In summary, we present data suggesting that the association of the rs26232 C allele with RA joint damage is mediated by higher tissue damaging activities of RASFs, and that this is not a mediated by levels of C5orf30 mRNA. A recognised limitation of the study is the reduced number of data points in certain assays. Due to the importance of matched patient RASF lines across assays and passage matching [38], it was not possible for all patient lines to be assayed for all experiments. We prioritised assays based on experimental evidence from previous research [3,5,15], and have been transparent in our data. It is possible that smaller genotype‒phenotype effects may not have been observed due to the reduced number of data points for some assays. It will be important to determine if a genotype‒phenotype association is also present in synovial macrophages, key cellular mediators in RA [4]; they also express C5orf30 at high levels and regulate anti-inflammatory and tissue-protective effects [6]. 

## Supplementary Materials

The following are available online at https://www.mdpi.com/2073-4409/8/10/1300/s1, Figure S1: rs26232 genotype is not associated with metalloproteases or macrophage chemokines by RASFs. Figure S2: rs26232 genotype is not associated with expression of adhesion markers or cathepsin K. Table S1: Sequences of oligonucleotide primers used in RT-PCR (5′–3′). Table S2: TaqMan Assay IDs used in RT-PCR.
