# Supplementary material for: Association of the Rheumatoid Arthritis Severity Variant rs26232 with the Invasive Activity of Synovial Fibroblasts
Source: Cells. 2019 Oct 22;8(10):1300. doi: 10.3390/cells8101300 (PMC6829881; doi:10.3390/cells8101300)
Supplement: Supplementary file 1 [file cells-08-01300-s001.zip › Supplementary-cells-587588-Figures-Revision.pdf]

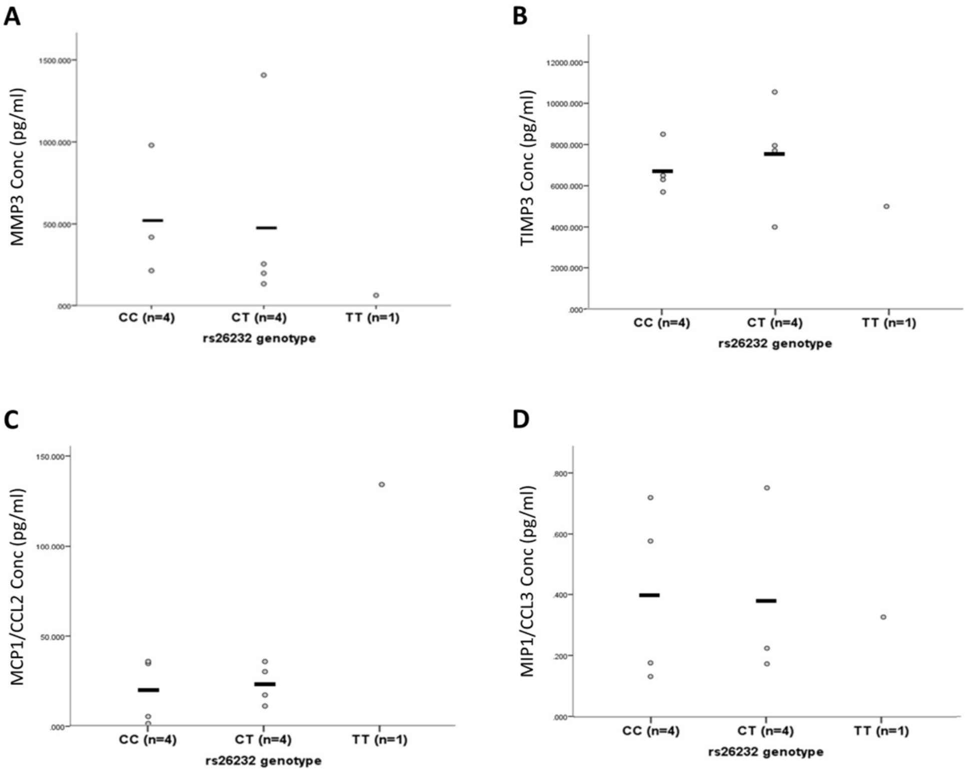

Figure S1: rs26232 genotype is not associated with production of metalloproteases or macrophage chemokines by RASFs. There was no association between rs26232 genotype and the production of (A) MMP3 (p=0.457), (B) TIMP3 (p=0.374), (C) MIP1 alpha (p=0.713) or (D) MCP1 (p=0.982). Each circle represents an individual donor. Horizontal black bars represent the cohort mean.

Supplementary Figure 2.

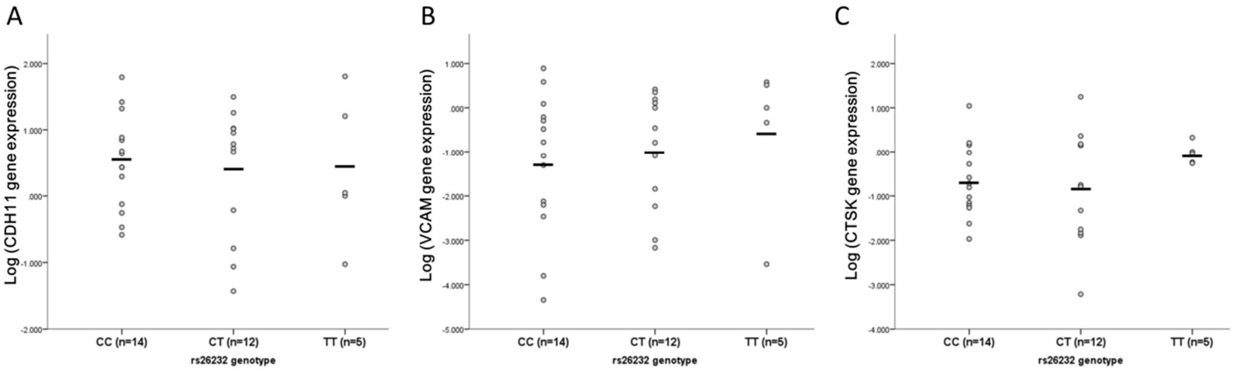

Figure S2: rs26232 genotype is not associated with expression of adhesion markers or cathepsin K. There was no association between rs26232 genotype and the production of (A) CDH11 gene expression (p=0.934), (B) VCAM (p=0.509), (C) CTSK (p=0.349). Each circle represents an individual donor. Horizontal black bars represent the cohort mean.
