# Supplementary material for: Association of the Rheumatoid Arthritis Severity Variant rs26232 with the Invasive Activity of Synovial Fibroblasts
Source: Cells. 2019 Oct 22;8(10):1300. doi: 10.3390/cells8101300 (PMC6829881; doi:10.3390/cells8101300)
Supplement: Supplementary file 1 [file cells-08-01300-s001.zip › Supplementary-cells-587588-Tables-revision.pdf]

Table S1. Sequences of oligonucleotide primers used in RT-PCR (5'-3').

	Forward Primer (5'-3')	Reverse Primer (5'-3')
**C5orf30**		
All transcripts	AGGACCGTGTCAGGCTACC	TGTTTGGAGCGTAAGGATGGC
Variant 1	GGACTTTGGGGCAGTACCTG	GAGTCCCAAGGGAACACAGAC
Variant 2	CCTGGGCCATGGTACCTGG	GAGTCCCAAGGGAACACAGAC
Variant 3	GGACTTTGGGGCAGTACCTG	GAGTCCCAAGGGAACACAGAC
**HPRT1**	ATGGACAGTGAACGTCTT	GAGTCCCAAGGGAACACAGAC

Table S2. TaqMan Assay IDs used in RT-PCR

	Assay ID	Probe Exon Location
**ICAM1**	Hs00164932_m1	exon boundary 2-3
**MMP14**	Hs00237119_m1	exon boundary 1-2
**CDH11**	Hs00901479_m1	exon boundary 4-5
**VCAM**	Hs01003372_m1	exon boundary 6-7
**CTSK**	Hs00166156_m1	exon boundary 7-8
**Eukaryotic 18S rRNA**	4310893E	n/a
